# Pulmonary alterations in cocaine users

**DOI:** 10.1590/S1516-31802004000100007

**Published:** 2004-01-08

**Authors:** Filho Mário Terra, Yen Chen Chin, de Paula Santos Ubiratan, Daniel Romero Muñoz

**Keywords:** Crack cocaine, Drug abuse, Dependency, Cocaine, Lung, Cocaína crack, Cocaína, Drogas de abuso, Abuso de cocaína, Transtornos relacionados ao uso de cocaína, Dependência de cocaína

## Abstract

**CONTEXT:**

Brazilian researchers have recently recognized a marked increase in the number of people using abusable drugs and the consequences of this habit. It has become a major public health problem in a potentially productive segment of the general population. In the last few years, several medical articles have given special emphasis to pulmonary complications related to cocaine use. This review is based on this information and experience acquired with groups of cocaine users.

**OBJECTIVE:**

To present to physicians the pulmonary aspects of cocaine use and warn about the various effects this drug has on the respiratory system, stressing those related to long-term use.

**DESIGN:**

Narrative review.

**METHOD:**

Pulmonary complications are described. These may include infections (Staphylococcus aureus, pulmonary tuberculosis, acquired immunodeficiency syndrome/aids, etc.), aspiration pneumonia, lung abscess, empyema, septic embolism, non-cardiogenic pulmonary edema, barotrauma, pulmonary granulomatosis, bronchiolitis obliterans and organizing pneumonia, pneumonitis and interstitial fibrosis, pneumonitis hypersensitivity, lung infiltrates and eosinophilia in individuals with bronchial hyperreactivity, diffuse alveolar hemorrhage, vasculitis, pulmonary infarction, pulmonary hypertension and alterations in gas exchange. It is concluded that physicians should give special attention to the various pulmonary and clinical manifestations related to cocaine use, particularly in young patients.

## RESUMO

**CONTEXTO:**

Recentemente, os pesquisadores brasileiros reconheceram que o número de pessoas que utilizam drogas de abuso e as conseqüências decorrentes desse hábito aumentaram consideravelmente, constituindo, hoje, um dos maiores problemas de saúde pública abrangendo uma camada de população potencialmente produtiva. Nos últimos anos, vários artigos médicos têm dado especial ênfase às complicações pulmonares relacionados ao uso de cocaína nos seus usuários. Com base nessas informações e na experiência adquirida com grupos de usuários de cocaína, redigimos esta revisão com o intuito de alertar a classe médica quanto aos diferentes efeitos no sistema respiratório.

**OBJETIVO:**

Apresentar aos médicos os aspectos pulmonares envolvidos com o uso da cocaína e alertálos quanto aos diversos efeitos dessa droga sobre o aparelho respiratório, ressaltando aqueles relacionados com o uso a longo prazo.

**TIPO DE ESTUDO:**

Revisão narrativa.

**MÉTODO:**

São descritas complicações pulmonares, tais como, infecções (Staphylococcus aureus, tuberculose pulmonar e extrapulmonar, síndrome da imunodeficiência adquirida, aids, etc.), pneumonia aspirativa, abscesso pulmonar, empiema, embolia séptica, edema pulmonar nãocardiogênico, barotrauma, granulomatose pulmonar, bronquiolite obliterante com pneumonia em organização, pneumonite e fibrose intersticial, pneumonite por hipersensibilidade, infiltrados pulmonares e eosinofilia em pessoas com hiper-reatividade brônquica, hemorragia alveolar difusa, vasculite, enfarto pulmonar, hipertensão pulmonar e alterações de troca gasosa. Assim, é necessário que os médicos atentem, hoje em dia, na sua rotina diária, para as diversas complicações pulmonares e manifestações clínicas associados ao uso de cocaína, particularmente nos jovens.

## INTRODUCTION

An illicit drug can be defined as any substance that is forbidden or not authorized by law and society's rules. Drug abuse refers to the consumption of licit or illicit drugs in order to attain an altered mental state.^1^ Thus, a drug is considered to be an abusable drug when it can be used for non-medical purposes and leads to addiction (compulsive use, loss of control over consumption and continued use in spite of social, economic or medical consequences). According to the World Health Organization (WHO), drug dependence corresponds to "the mental state, and often a physical state, resulting from the interaction between a living organism and a drug". It is characterized by behavior that always includes the compulsion to take the drug in order to experience its psychic effect and sometimes to avoid the discomfort of its absence.^2^

Drug abuse has reached considerable proportions in the last few years,^3^ and currently ranks as a major public health problem. The use of illegal drugs has spread to potentially productive segments of the general population, predominantly young^4^ adult men.^5-7^ In Brazil, surveys carried out by Cebrid (Brazilian Information Center on Psychotropic Drugs),^8,9^ Grea (Interdisciplinary Study Group on Alcohol and Drugs)^10^ and Denarc (State Department of Narcotics Investigations)^11^ have shown increasing consumption of such drugs, particularly of cocaine. In other studies involving Brazilian students,^12,13^ it was shown that 90% of the participants had already tried some illegal drug at least once, such drugs were predominantly used by men and consumption of abusable drugs had begun at an early age, around 10-12 years old.^14^ Among the abusable drugs, alcohol, marijuana and cocaine are the ones most consumed worldwide, in the salt or base forms.^14,15^ Most users take them simultaneously or report previous use of other drugs. In general, cocaine is used with alcohol.^7,16-19^ Interaction between alcohol and cocaine results in the formation of cocaethylene,^20^ which can increase the effects and toxicity of cocaine.^16^ Some authors have reported that ethanol and cocaine association increases the risk of sudden death.^21, 22^

Muñoz et al.^23^ studied the use of alcohol, marijuana and cocaine in 63 victims of violent death in the city of Guarulhos (state of São Paulo, Brazil). Blood and urine samples obtained during autopsy were used in the study, and 61.9% of the victims were positive for the three drugs. Alcohol was detected in 53.33%, cocaine or its metabolites were found in 25.86% and cannabinoids in 17.24% of the cases. In an analysis of hair samples from 38 victims of the same group with positive results for cocaine in urine, Toledo et al.^24^ found this drug or its metabolite (benzoylecgonine) in all samples.

Cocaine is an alkaloid found in the leaves of *Erythroxylon coca*, commonly known as coca, a native plant of the Andean and Amazon regions in South America. It was first extracted and identified by the German chemist Albert Niemann in the mid-19th century.^25^ The inhabitants of the Andes chewed the leaves to ward off fatigue, hunger and thirst, to enhance endurance, and to promote a sense of wellbeing. Because of its pharmacological properties, it had medical use as a tonic/elixir for several diseases and also as local anesthetic in eye, ear and throat surgery.^1^ It was subsequently replaced by synthetic anesthetics such as lidocaine.^26^ Cocaine was also used in popular beverages and the most popular of these were Vin Mariani and Coca-Cola in its original formulation.^1^ It has no therapeutic indication today and its importance derives from being considered to be an abusable drug.

Cocaine is a powerful central nervous system stimulant and has very complex activity, both through central and peripheral pathways. Its anesthetic effect is achieved by blocking sodium influx in the nerve cell membrane, thereby preventing nervous transmission.^25,27,28^ It is assumed that alterations in dopamine transport are the mechanism for the euphoric and compulsive effects of cocaine.^29-33^

This illicit drug is sold on the street, mixed with similar-looking substances like lidocaine, talcum powder or sugar. Cocaine is generally consumed in freebase form or smoked as "crack". It can be absorbed via intranasal, intravenous or vaginal routes in the hydrochloride form or by massaging the gums in coca paste form (prepared using solvents like kerosene and gasoline). It can be smoked either in pipes or cigarettes.^1^

"Crack"^34,35^ was developed in the second half of the 1980s, when the characteristics of cocaine were altered so that it could be smoked. Crack is prepared by heating and evaporating the hydrochloride with a base (generally sodium bicarbonate). After cooling, irregular crystals or "rocks" are the final result. These produce a cracking sound when heated.^33,36^ Thus, it became known as "crack"^5^ and has become the most popular form for consumption of this drug. Cocaine obtained by this process may be 100% pure, but the purity can be as low as 20% because contaminants are not always removed and the drug is often further adulterated by the producer.^6^

### Pulmonary effects of cocaine

There are several organic consequences (Table 1) resulting from the chronic use of cocaine by different administration routes and its local or systemic pharmacological effects. Among these are adverse effects on the lungs.^37^ Respiratory system alterations depend not only on the administration route, but also on the dose and time of use, presence of microbiological contaminants or substances used to adulterate the sample, sharing of paraphernalia used for the drug consumption and host responses of the individual user.^38^ Small doses of cocaine cause acute respiratory stimulation and large doses lead to respiratory depression. In some users, however, cocaine can promote an undesirable paradoxical effect with an initial increase in respiratory rate and depth followed by depression.^2^

**Table 1:** Pulmonary effects of cocaine

Acute respiratory symptoms
Barotrauma and bullous emphysema
Pulmonary edema
Pulmonary hypersensitivity reaction (bronchospasm)
Alveolar hemorrhage
Alveolitis, pneumonitis and interstitial fibrosis
Bronchiolitis obliterans with organizing pneumonia
Vasculitis and pulmonary hypertension

When crack is smoked, the lungs are directly exposed to the volatile drug and to the products resulting from its combustion. Rapidly absorbed by the mucous membrane and alveolar epithelium, the drug reaches the bloodstream almost immediately, providing the user with a more rapid and intensive physiological and psychological effect than is obtained by intravenous injection.^32,39^

Smoking cocaine can produce acute lung injury, and several hypotheses have been proposed to explain this. These include cell anoxia resulting from the cocaine-induced lung vasoconstriction; direct toxic effect on the alveolar capillary endothelium and bronchial epithelium; and hypersensitivity reaction to basic cocaine components.^33,36,40^

Several pulmonary complications related to cocaine use (Table 2) and administration route have been reported in the literature. Opportunistic and secondary systemic infections with pulmonary involvement have been reported (*Staphylococcus aureus*, pulmonary tuberculosis or aids^33,41^ due to poor cleaning of the paraphernalia used),^33^ aspiration pneumonia and lung abscess, empyema and pulmonary granulomatosis.^6,42-44^ Thrombophlebitis and septic endocarditis with release of emboli, septic emboli, pulmonary artery obstruction, mycotic aneurysms (pulmonary artery) and lung infiltrates have been described in relation to intravenous cocaine use.^38^

**Table 2:** Pulmonary histopathology features related to cocaine use*

**Alveolar**	
	Pulmonary edema
	Focal hemorrhages
	Intact alveolar erythrocytes
	Macrophages with hemosiderin
	Foreign material in the alveoli
	Thickening of the alveoli walls (pneumoconiosis-like)
	Type II pneumocyte hyperplasia
	Blebs
	Emphysema
**Bronchiolar**	
	Bronchiolitis obliterans and organizing pneumonia
**Bronchial**	
	Alterations of the bronchial epithelium
	Thickening of the bronchial wall
	Bronchitis
**Interstitial**	
	Granuloma of the giant cell type (injection)
	Birefringent crystals in the macrophage (around vessels or airways)
	Interstitial fibrosis
**Vascular**	
	Vascular bullous (injection)
	Septic emboli (injection)

* *According to the literature.^38,53,75,76,77^*

#### Barotrauma and bullous emphysema

Pneumothorax and pneumomediastinum^1,38,45-48^ are commonly related to cocaine inshalation or smoking, in the freebase or crack forms. These complications result from coughing or intense Valsalva's maneuver performed to heighten the effect of the drug, thereby leading to a sudden increase in intrabronchial and intraalveolar pressure with subsequent alveolar rupture and air penetration in the pulmonary interstitium.^41^ The free air can dissect the peribronchial connective tissue in the mediastinum, pericardium, pleural cavity and subcutaneous tissues, resulting in pneumopericardium^48^ and subcutaneous emphysema.^49^ Gurney and Bates^50^ reported the occurrence of bullous emphysema in 10 drug users: they were all young, smokers (3 of them for less than 3 years), with normal alpha-1 antitrypsin serum levels.

#### Pulmonary edema and immune-mediated pulmonary disease

Reports in the literature describe noncardiogenic pulmonary edema in cocaine users, especially after crack smoking^38,51-53^ Although the precise mechanism is unclear, some authors believe that there is a relationship with increased lung capillary permeability, since bronchoalveolar lavage fluid from cocaine users has shown increased protein concentration.^51^

There are reports^53-55^ in the literature suggesting that crack users can develop pulmonary hypersensitivity reaction with bronchospasm exacerbation.

#### Alveolar hemorrhage

Alveolar hemorrhage has been described in the last ten years as one of the pulmonary complications caused by cocaine use.^38,41,56-58^ In 1988, Murray et al.^59^ reported the first case of alveolar hemorrhage and hypoxemic respiratory failure associated with freebase cocaine smoking, and open-chest biopsy revealed diffuse alveolar hemorrhage. The two cases published by Bouchi et al.^60^ had hemoptysis and dyspnea symptoms, and alveolar hemorrhage was seen via bronchoscopy as blood originating from the bronchial openings, and via bronchoalveolar lavage as hemosiderin in alveolar macrophages. This last paper suggested that cell damage caused by pulmonary vasoconstriction and alveolar cells caused directly by the drug are the two mechanisms that could possibly trigger hemorrhage.^60^

In reviewing histopathological lung sections obtained during the necropsy of 52 cadavers with positive toxicological tests for cocaine (equal to or higher than 0.01 mg/dl) in blood, urine or bile, Bailey et al.^61^ found both acute and chronic alveolar hemorrhage (58 and 40% of the cases, respectively), pulmonary vascular congestion (88%), alveolar edema (77%) and foreign material and microgranulomas (11%).

#### Alveolitis, pneumonitis and interstitial fibrosis

Ongoing use of cocaine by inhalation can lead to pneumonitis^62^ and interstitial fibrosis.^41,52,53,62^ In their histopathological study, Bailey et al.^61^ found pneumonitis characterized by thickening of the alveolar septum and infiltrate of neutrophils, lymphocytes, macrophages, and eosinophils, and interstitial fibrosis evidenced by type II pneumocyte hyperplasia.

Other authors^63^ found extensive interstitial inflammatory infiltrate in the lungs of 14 out of 17 forensic necropsies performed on drug users. Their other findings were acute inflammatory alterations of alveolar walls with the presence of exsudate and cells in the alveoli, and vascular obstruction possibly caused by injection of mixtures for oral use (intravenous injection of adulterated cocaine). According to Oubeid et al.,^44^ talcum powder is the agent most often used to adulterate the drug and this can cause pulmonary fibrosis.

#### Bronchiolitis obliterans with organizing pneumonia

Reports in the literature accept that the bronchiolitis obliterans with organizing pneumonia observed in some freebase cocaine users may be the result of a direct toxic effect of the drug on the airways.^41,49,64^ It is also suggested that bronchiolitis obliterans with organizing pneumonia can occur as an idiopathic response to cocaine or substances used in its adulteration, or even from contaminants found in the paraphernalia used for smoking cocaine.^64^

#### Vasculitis and pulmonary hypertension

Vasculitis associated with abusable drugs is well-documented in the literature, and some reports have described it as necrotizing vasculitis^65^ that is similar to polyarteritis nodosa.^66^ The mechanism by which cocaine causes vascular pathology remains unknown, but it has been associated with cerebral vasculitis.^67-70^

Although there is mention in the literature that long-term use of intravenous cocaine is associated with pulmonary hypertension, very few cases have been reported and effectively proven.^42,71-74^ Yakel and Eisenberg^71^ used transthoracic echocardiography to estimate the pulmonary artery pressure in 13 asymptomatic long-term users of intravenous cocaine, aged between 20 and 45 years, and obtained high pulmonary artery pressure (over 30 mmHg) in eight of these subjects. It is believed that such a pressure increase may be due to a reduction in the pulmonary vascular bed caused by the granulomatous process^44^ that results from the addition of insoluble agents to the illicit drug (especially talcum powder, cellulose and starch). These agents generate a local chronic interstitial inflammatory process or microembolization in pulmonary arterioles and capillaries.

#### Clinical manifestations

The respiratory symptoms are varied and are either nonspecific or directly related to the pulmonary disease (Table 3). According to the literature, respiratory symptoms may appear a couple of hours after cocaine use, although in some cases they may occur within a few minutes. The most common clinical complaints related to lung involvement are chest pain^41-47^ (pleuritic or non-pleuritic); dyspnea;^46,47,53,57,64,77,78^ cough^6,34^ with expectoration of dark material (inhalation of residual combustion products) or blood material; hemoptysis^42,53,57,64,77^ (diffuse alveolar hemorrhage);^41,57^ fever;^47,53,79^ and asthma and recurring bronchospasm episodes (exacerbated or non-exacerbated).^41,49,54,55^ Such symptoms have been reported mainly among crack users^6,80^ and not with inhalation or intravenous use of cocaine. According to Suhl and Gorelick^81^ and Delbono et al.,^82^ the prevalence of such symptoms is highly variable: cough was reported in 26% and 61%, and dyspnea in 21% and 44%.

**Table 3:** Clinical manifestations related to cocaine*

Chest pain
Dyspnea
Cough
Hemoptysis
Fever
Bronchospasm

* *Mainly in "crack" users.*

#### Pulmonary function tests

There are very few studies related to pulmonary function in cocaine users. A study performed in 1987 indicated that smoking cocaine could modify the forced expiratory flow.^77^ Later studies, however, involving long-term cocaine users smoking freebase, showed spirometry with normal results in most of them,^40,81,83^ with no evidence of obstructive or restrictive processes and association with nonspecific airway hyper-reactivity.^80^

Only three of the several studies among crack users have shown decreased carbon monoxide diffusion capacity.^40,83,84^ The authors of these studies even considered the possibility that this alteration could be related to the alveolar-capillary membrane or pulmonary vasculature lesion. Tashkin et al.,^77^ on the other hand, did not observe any diffusion alteration among a cohort of 16 cocaine smokers (moderate users), who also used marijuana but did not use tobacco and/or intravenous drugs. According to Tashkin, in another paper published later, discrepancies in the diffusing capacity observed in other studies may be due to sample size; frequency, intensity or duration of cocaine smoking; effects from other drugs smoked; complications resulting from the use of intravenous drugs; or characteristics of the crack users themselves.^85^

Recently, Goldbaum et al.^86^ performed a study in Brazil on pulmonary function in 28 cocaine users, all male, with ages ranging from 18 to 50 years. Four of the subjects were also tobacco users and 24 mentioned marijuana and tobacco use. All subjects had respiratory symptoms (cough, expectoration of dark sputum, wheezing and dyspnea) during or after cocaine use, and had normal chest radiographs. One man presented micronodules and septal thickening compatible with respiratory bronchiolitis via high-resolution computed tomography scan. The spirometry values were normal, but mild alterations in carbon monoxide diffusing capacity (60-80% of predicted value) were detected in 7 subjects. The results obtained in that study are compatible with others in the literature.

#### Radiological findings

The radiological findings reported among cocaine users include atelectasis,^47^ alveolar opacification (alveolar hemorrhage)^38,59^ that can regress within a week,^41^ bilateral interstitial and alveolar infiltrates^51,52^ (noncardiogenic pulmonary edema), transient^53^ pulmonary infiltrates,^79^ pneumonia, pulmonary nodules,^64^ interstitial infiltrate and fibrosis. In crack users, however, the radiological abnormalities usually described are pneumothorax and pneumomediastinum.^48,78^ Pneumopericardium,^48,87^ hemopneumothorax^47^ and bullous emphysema^50^ (intravenous administration) have also been reported, especially in the upper lobes.

#### Chest computerized tomography (CT scan)

Yen et al.^88^ described computed tomography scan and pathological findings from three patients who were tobacco smokers and frequent users of marijuana and especially cocaine (powder and/or crack). Their radiography was normal but high-resolution computed tomography scan showed a pattern suggesting septal thickening and diffuse micronodules. Pathological findings of transbronchial biopsy were compatible with desquamative pneumonia associated with respiratory bronchiolitis. On the basis of the clinical history and computed tomography and pathological findings, the authors suggest that the use of cocaine, marijuana and/or tobacco in association can cause pulmonary alterations that may progress with long-term illicit use.^88^

#### Scintigraphy

To evaluate the alveolar-capillary membrane integrity among crack users, Susskind et al.^89^ used lung clearance with ^99m^Tc-labeled DTPA (diethylenetriamine pentaacetic acid) in 23 subjects: seven used only crack, seven were tobacco and crack users, and nine used crack and marijuana simultaneously. The authors believe that the pulmonary epithelium lesion and consequent increase in lung permeability result from the direct action of crack vapors or pyrolysis products and the release of inflammatory mediators by effector or structural cells exposed to smoking.^89^ However, Tashkin et al.^90^ reported different results in another study. They believe that discrepancies found between the two studies are due to the previous smoking history and other exposure characteristics in the two populations of crack smokers studied.^90^

Terra-Filho et al.^91^ studied Ga-67 lung scintigraphy in a group of 26 tobacco smokers, all with a history of illicit drug use (cocaine or cocaine and marijuana) and 22 reporting simultaneous use of marijuana and cocaine. The Ga-67 scintigraphy was positive in eight subjects, but lung parenchyma uptake in the control group subjects (tobacco smokers) was negative. Both groups had normal chest X-rays. The authors believe that the alterations in the Ga-67 scintigraphy studies detected in the illicit drug user group suggest lung inflammatory disease preceding the X-ray abnormalities.^91^

### Bronchoalveolar lavage and sputum

In analyses of bronchoalveolar lavage among cocaine smokers, several authors have reported the presence of large numbers of eosinophils, Charcot-Leyden crystals,^38^ and larger populations of alveolar macrophages, often containing hemosiderin, and increased protein concentrations^38,79^ (higher than in congestive heart failure),^51^ which were greater than what was found in bronchoalveolar lavage among subjects that did not use this drug^92^ According to the same authors, these findings would represent not only higher alveolar epithelium permeability^38,51^ but also probable interstitial alveolar inflammatory response to the aggression to the lungs from the drug^64,92^

Other reports^36,93,94^ mention that the alveolar macrophages recovered produced fewer inflammatory cytokines such as IL-6, IL-8 and tumor necrosis factor alpha, but produced larger amounts of the immunosuppressor factor TGF-beta (transforming growth factor beta) than from macrophages from non-cocaine smokers.^36^ This indicates that chronic use of cocaine can limit lung response, thus making the user more susceptible to infections.

## CONCLUSION

Whatever the form and administration route used, cocaine causes deleterious effects in the lungs. The respiratory symptoms are not specific, but carbonaceous sputum (dark material) is a characteristic of crack cocaine users. For the majority of long-term addicted subjects, pulmonary function tests show normal results with no evidence of obstructive or restrictive disturbances, but it is possible to identify pulmonary hypersensitivity reaction (bronchospasm) from such procedures when associated with clinical signs. Radiological findings are very important for establishing diagnosis of pulmonary complications in cocaine users (alveolar hemorrhage, pulmonary edema and fibrosis) and crack addicts (pneumothorax and/or pneumomediastinum). High-resolution computed tomography increases the sensitivity for detection of interstitial fibrosis, pneumonitis, alveolitis and bronchiolitis obliterans with organizing pneumonia, but the cost will restrict its use to individual cases with specific complaints. The principal noninvasive technique for investigating pulmonary hypertension is echocardiography. Scintigraphic evaluation of illicit drug users is expensive and is used only for academic purposes. Nowadays, bronchoalveolar lavage is used as a clinical tool for evaluating cocaine users only to rule out other diagnoses, and for research.

The diagnosis of illicit drug-induced lung disease may be difficult if an accurate history of drug use is not obtained. Establishing the diagnosis of drug-induced lung disease is important, because the appropriate treatment may also include referral to drug counseling or rehabilitation.
